# Dietary phosphate exacerbates intestinal inflammation in experimental colitis

**DOI:** 10.3164/jcbn.16-117

**Published:** 2017-07-28

**Authors:** Kohei Sugihara, Masashi Masuda, Mari Nakao, Maerjianghan Abuduli, Yukiko Imi, Naoko Oda, Toshiya Okahisa, Hironori Yamamoto, Eiji Takeda, Yutaka Taketani

**Affiliations:** 1 Departments of Clinical Nutrition and Food Management, Institute of Biomedical Sciences, Tokushima University Graduate School, Tokushima 770-8503, Japan; 2 Department of Gastroenterology and Oncology, Institute of Biomedical Sciences, Tokushima University Graduate School, Tokushima 770-8503, Japan; 3 Department of Health and Nutrition, Faculty of Human Life, Jin-ai University, Fukui 915-8586, Japan

**Keywords:** dietary Pi, DSS-induced colitis, proinflammatory cytokine, NF-κB, ROS

## Abstract

The recent widespread consumption of Western diets and food additives worldwide is associated with excessive inorganic phosphate intake. However, researchers have known little about the impact of dietary phosphate intake on the development of inflammatory bowel disease to date. In this study, we investigated the effects of dietary phosphate on intestinal inflammation in experimental colitis. Sprague-Dawley rats were fed different phosphate diets (0.5%, 1.0% and 1.5% phosphate) with or without dextran sulfate sodium. For *in vitro* study, the effects of phosphate on proinflammatory cytokine induction and reactive oxygen species production in RAW264.7 macrophage were examined. Dietary phosphate exacerbated intestinal inflammation in experimental colitis in a dose-dependent manner, as assessed by the clinical disease activity score, colon length, and histology. Furthermore, the high phosphate diet increased myeloperoxidase activity and proinflammatory cytokine mRNA expression through the activation of nuclear factor κB in the inflamed colon. In addition, high phosphate loading in RAW264.7 cells directly enhanced reactive oxygen species production and proinflammatory cytokine gene expression. Our results demonstrated that the high phosphate diet exacerbated intestinal inflammation in experimental colitis. These findings have important therapeutic implications for inflammatory bowel disease patients.

## Introduction

Inflammatory bowel disease (IBD) is a term describing a group of chronic recurrent inflammatory disorders of the gastrointestinal tract, typified by Crohn’s disease and ulcerative colitis. The exact pathogenesis of IBD is complex and involves genetic factors, the host immune system, and environmental factors.^(1)^ With environmental factors, the variety of nutrients is a major factor affecting the intestinal inflammation and microbiota, and diet has been implicated in the development of these diseases.^(2)^ Several diets—such as high intake of refined sugar-containing food, fast foods, and red and processed meat—have been identified as risk factors for the onset and relapse of IBD.^(3–5)^ In particular, fast foods and processed meat contain high fat levels, which results in exacerbation of intestinal inflammation, but these foods also frequently contain a large amount of inorganic phosphate (Pi) as a food additive.

Pi is an essential nutrient for skeletal formation, energy metabolism, and intracellular signaling, and is mainly supplied from the diet including meat, grains, and dairy products.^(6)^ Furthermore, Pi is the main component of many food additives which are utilized for various purposes. Although there is little clear evidence to shows increasing Pi intake in the general population, excessive consumption and widespread use of Pi is currently raise a public health concern.^(7,8)^ Indeed, recent epidemiological studies have suggested that high dietary Pi intake is associated with increased cardiovascular risk and all-cause mortality.^(9,10)^ Moreover, high Pi loading in the body can cause a wide range of cellular and tissue injuries.^(11–15)^ This Pi cytotoxicity due to excessive retention of Pi was shown to be associated with increased mortality and cardiovascular complication in patients with chronic kidney disease (CKD).^(16,17)^ Thus, dietary Pi restriction and Pi binder administration are beneficial treatments for CKD patients to prevent the progression of diseases and cardiovascular complications.

Along with other researchers, we recently reported that Pi metabolism was altered by lipopolysaccharide (LPS)-induced systemic inflammation.^(18)^ whereas Pi overloading activated nuclear factor κB (NF-κB) signaling via reactive oxygen species (ROS) production,^(15)^ thus suggesting an interactive association between Pi and inflammation. These interactions between serum Pi levels and serum inflammatory markers are also observed in CKD patients.^(19)^ Furthermore, low-Pi diet and Pi binder treatment ameliorated systemic inflammation in CKD model rat,^(14)^ and thus excess Pi intake was linked to inflammation; itself an important potential mediator of inflammatory disease.

Increasing evidence from both clinical and animal studies suggests that Western diets aggravate intestinal inflammation. However, several reviews of dietary management in IBD patients did not mention dietary Pi intake, and little is known about the impact of dietary Pi on intestinal inflammation.

In this study, we examined the effects of dietary Pi on intestinal inflammation using an experimental colitis rat model. We demonstrated that high Pi diet exacerbated intestinal inflammation in experimental colitis, and Pi loading directly enhanced ROS production and proinflammatory cytokines production via NF-κB activation in RAW264.7 macrophages.

## Materials and Methods

### Animal and experimental design

Seven-week-old male Sprague-Dawley rats were purchased from Japan SLC (Shizuoka, Japan) and individually caged in a climate-controlled room (22 ± 2°C) with a 12-h light:12-h dark cycle. All rats were randomly divided into 6 groups and fed different Pi diets (0.5%, 1.0% and 1.5% Pi) with or without dextran sulfate sodium (DSS). In this study, the 0.5% Pi diet was designated as the control diet according to the previous studies.^(20,21)^ The diets were based on modified AIN-93G rodent diet, formulated with casein and K_2_HPO_4_ (Table 1). Rats were fed different Pi diets for 7 days before DSS treatment, and then given 2.5% DSS [molecular weight (MW): 36,000–50,000; MP Biomedicals, Illkirch-Graffenstaden, France] orally in drinking water for 7 days. Changes in body weight and characteristics of stool were observed during the DSS treatment period, and the rats were then euthanized under anesthesia at day 7 for evaluation of colitis severity. The disease activity index (DAI) score was assessed using the method described previously.^(22)^ Distal segments of colon were sampled for histological evaluation, and adjacent segments of colon were taken for analysis of gene and protein expression and myeloperoxidase (MPO) activity. The experiments were approved by the Animal Care and Use Committee of Tokushima University and were performed in accordance with the guidelines for the care and handling of laboratory animals.

### Histological analysis

Colonic tissues were fixed with 4% paraformaldehyde, embedded in paraffin, and cut into slices 5 µm thick. Colonic sections were deparaffinized, rehydrated, and stained with hematoxylin and eosin for routine histology, or with Alcian blue and periodic acid–Schiff (AB/PAS). All histological quantitation was performed blinded using the scoring system previously described.^(23)^ The scoring system includes severity of inflammation (0–3: none, slight, moderate, severe); extent of injury (0–3: none, mucosal, mucosal and submucosal, transmural); and crypt damage (0–4: none, basal 1/3 damaged, basal 2/3 damaged, only surface epithelium intact, entire crypt and epithelium lost). The score of each score was multiplied by a factor reflecting the percentage of tissue involvement (×1: 0–25%, ×2: 26–50%, ×3: 51–75%, ×4: 76–100%).

### Measurement of myeloperoxidase (MPO) activity

 Colonic MPO activity was measured according to the method shown in a previous report.^(24)^ Briefly, colonic tissues were homogenized using a Polytron homogenizer in ice-cold 50 mM potassium phosphate buffers (pH 6.0) containing 0.5% hexadecyltrimethylammonium bromide. The homogenate was then sonicated for 10 s, freeze-thawed 3 times, and centrifuged at 20,000 *g* for 30 min at 4°C. The supernatant was added in 50 mmol/L phosphate buffer (pH 6.0) containing 0.167 mg/ml O-dianisidine hydrochloride and 0.0005% hydrogen peroxide. The kinetics of absorbance at 450 nm was measured using a spectrophotometer at 25°C. Protein concentration of the supernatant was determined using a Bradford assay kit (Bio-Rad Laboratories, Hercules, CA) for calibration. MPO was expressed in units per mg of protein; 1 unit of MPO activity was defined as degrading 1 µmol hydrogen peroxide/min at 25°C.

### Cell culture and treatment

The RAW264.7 murine macrophage cell line was kindly provided by Prof. T. Nikawa (Tokushima University) and were cultured medium consisted of Dulbecco’s modified Eagles medium (DMEM; Sigma-Aldrich, St. Louis, MO) supplemented with 10% fetal bovine serum (Biowest, Nuaillé, France), 100 µg/ml streptomycin, and 100 U/ml Penicillin (Sigma-Aldrich). The RAW264.7 cells were grown in 100 mm tissue culture dishes to 70–80% confluence and cultured at 37°C in a humidified atmosphere containing 5% CO_2_. The DMEM contain 0.9 mM phosphate in itself and, RAW264.7 cells were treated with appropriate amounts of sodium phosphate buffer (0.1 M Na_2_HPO_4_/NaH_2_PO_4_, pH 7.4) to produce final Pi concentrations.

### RNA extraction and real-time reverse transcription polymerase chain reaction (RT-PCR)

Total RNA was extracted from colonic mucosa using an RNeasy plus mini kit (Qiagen GmbH, Hilden, Germany) and from RAW264.7 cells using TRIzol Reagent. We purified RNA using the lithium chloride procedure described previously,^(25)^ since DSS is known to interfere with PCR reactions. The cDNA was synthesized using a reverse transcriptase kit (Invitrogen) with an oligo-dT primer. After cDNA synthesis, real-time RT-PCR (Applied Biosystems, Carlsbad, CA) was performed using SYBR Green PCR master mix (Thermo Fisher Scientific, Waltham, MA). The amplification programs were set as follows: initial denaturation at 95°C for 10 min, followed by 40 cycles of 95°C for 10 s, 60°C for 15 s, and 72°C for 15 s. The primer sequences are shown in Table 2. All sample mRNA levels were normalized to GAPDH for animal or β-actin for RAW264.7 cells and the relative mRNA levels were calculated.

### Nuclear protein extraction and electrophoretic mobility shift assay

Nuclear protein was extracted using buffer A [10 mM HEPES-KOH (pH 7.8), 0.1 mM EDTA, 5 mM KCl, 0.1% NP40] containing 0.5 mM PMSF, 1 mM DTT, and protease inhibitor. After centrifugation at 9,100 *g* for 1 min at 4°C, the supernatant was collected as a cytosol extract. The nuclear pellets were washed 3 times with buffer A and resuspended in buffer C [50 mM HEPES-KOH (pH 7.8), 0.1 mM EDTA, 420 mM KCl, 5 mM MgCl_2_, 20% glycerol]. After centrifugation at 12,700 *g* for 15 min at 4°C, the supernatant was collected as a nuclear extract. Electrophoretic mobility shift assay (EMSA) analysis was performed as previously described.^(26)^ Briefly, purified DNA fragments were radiolabeled with [γ-^32^P] ATP (110 TBq/mmol; ICN, Costa Mesa, CA) using T4 polynucleotide kinase (Takara, Shiga, Japan). Prepared nuclear protein were incubated with the radiolabeled probe in binding buffer [10 mM Tris/HCl, pH 7.5, 1 mM dithiothreitol, 1 mM EDTA, 10% glycerol, 1 mM MgCl_2_, 0.25 mg/ml BSA, 2.5 µg/ml salmon sperm DNA and 2 µg poly(dI-dC) (dI-dC) (GE Health Care, Tokyo, Japan)] in a final volume of 20 µl for 30 min at room temperature (20°C). NF-κB consensus oligonucleotides sequence was purchased from Santa Cruz Biotechnology (Dallas, TX; catalogue number sc-2505). The reaction mixture was then subjected to electrophoresis on a 5% polyacrylamide gel with 0.1× TBE running buffer for 1.5 h at 150 V. The gel was dried and analyzed with a Bio-imaging analyzer (FLA-9000; GE Health Care).

### Immunofluorescence staining

Colonic sections were deparaffinized, rehydrated, and microwaved in 10 mmol/L sodium citrate (pH 6.0) for 5 min to retrieve antigen. After a blocking step with blocking one histo (Nacalai Tesque, Kyoto, Japan) for 10 min at room temperature, slides were incubated with primary antibodies for NF-κB p65 (1:400, Santa Cruz Biotechnology) overnight at 4°C. Subsequently, they were washed in phosphate-buffered saline (PBS) and incubated with Alexa Fluor 555-conjugated secondary antibodies (Molecular Probes, Eugene, OR) for 1 h at room temperature. The slides were then counterstained with 4',6-diamidino-2-phenylindole (DAPI; Sigma) to stain nuclei. RAW264.7 cells were cultured on glass cover-slips in 12-well plates *in vitro*. After the indicated treatment, the cells were washed with cold PBS twice, fixed with 4% paraformaldehyde for 15 min, and permeabilized with 0.1% Triton X-100 in PBS for 5 min. The cells were then blocked in 0.8% bovine serum albumin for 30 min and incubated with primary antibody (NF-κB p65, 1:400) for 1 h at room temperature. After being washed 3 times, secondary antibody-(conjugated with Alexa Fluor 568 and DAPI) was stained for 1 h at room temperature. Fluorescence was visualized using a BZ-9000 fluorescence microscope (Keyence, Osaka, Japan).

### Western blot analysis

The nuclear and cytosol extract were mixed with sample buffer, incubated at 95°C for 5 min in the presence of 2-mercaptoethanol, and subjected to sodium dodecyl sulfate polyacrylamide gel electrophoresis (SDS-PAGE). Following electrophoresis, the proteins were transferred to a polyvinylidene difluoride membrane (Immobilon-P; Merck Millipore, Darmstadt, Germany). The membranes were blocking with 5% skim milk and treated with primary antibody for NF-κB p65 (1:1,000), Lamin B (1:1,000, Santa Cruz Biotechnology), and β-actin (1:2,000, Sigma) overnight at 4°C. The membranes were then incubated with horseradish peroxidase-conjugated secondary antibody. The blots were visualized with the enhanced chemiluminescence (ECL) reagents.

### Measurement of ROS production

We measured ROS production using the aminophenyl fluorescein (APF; Goryo Kagaku, Hokkaido, Japan) indicators, which can specifically recognize many types of ROS.^(27)^ RAW264.7 cells were placed on a 96-well plate in 200 µl medium. The following day, cells were rinsed with modified Hank’s balanced salt solutions (HBSS) and incubated with 20 µM APF (diluted in HBSS) for 30 min at 37°C. After incubation with APF, cells were treated by HBSS containing different concentration of Pi with or without specific oxidase inhibitors for 120 min. Fluorescence intensity at excitation 490 nm and emission 515 nm was measured by a fluorescent plate reader.

### Statistics

Data are expressed as mean ± SEM. Statistical analysis was performed using unpaired *t* test for two-group comparisons and one-way ANOVA with Tukey post-hoc test for multigroup comparison. All data analysis was performed using GraphPad Prism 5 software (Graphpad Software, San Diego, CA). *P*<0.05 was considered to indicate statistical significance.

## Results

### Effects of dietary Pi on clinical symptoms in DSS-induced colitis

**Fig. 1:**
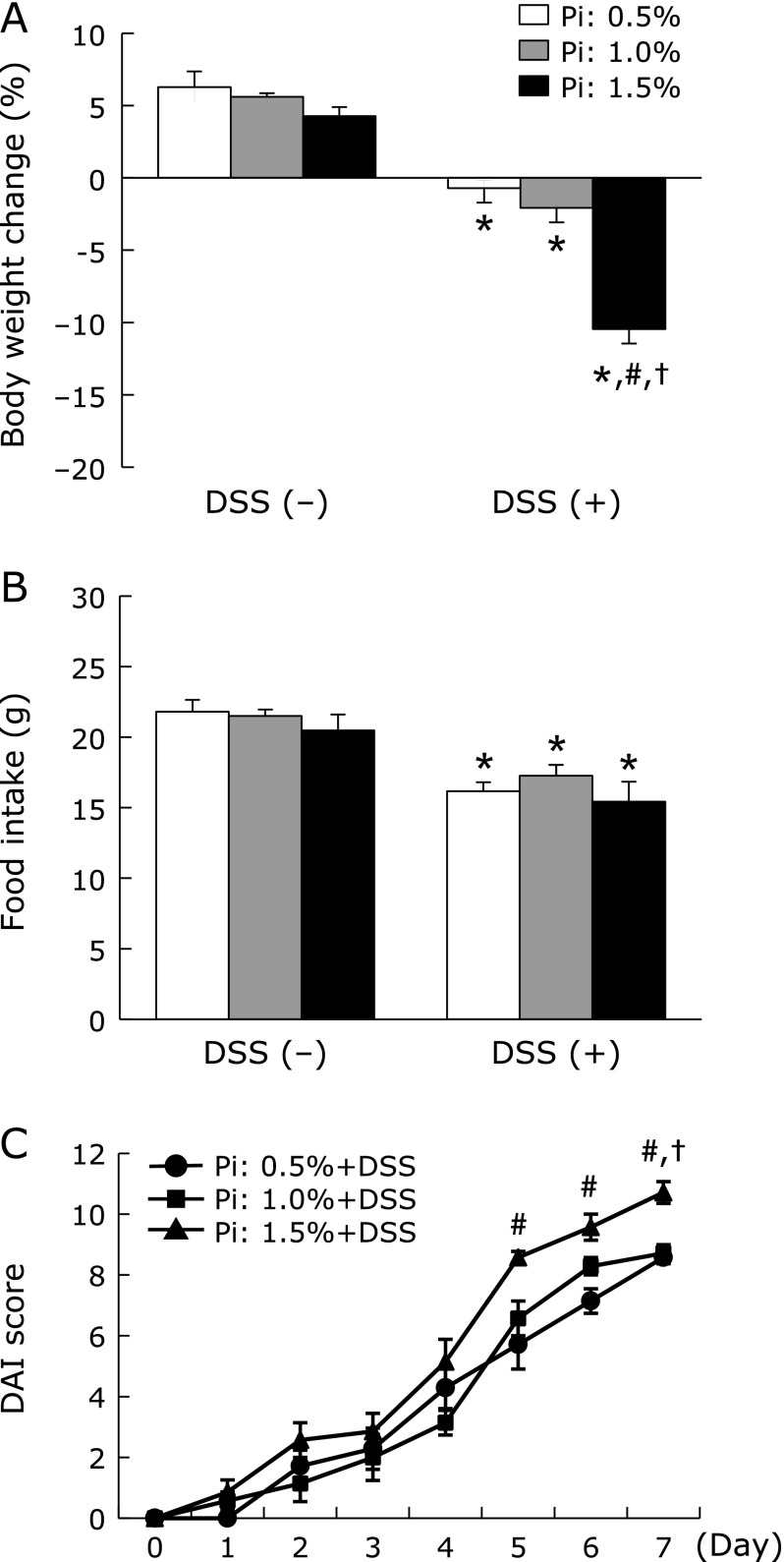
Increase in dietary Pi intake exacerbated clinical symptoms in DSS-induced colitis. (A) Changes in body weight during DSS-treated periods. (B) Food intake during DSS-treated periods. (C) Changes in DAI score calculated by summing the means of 3 scores (stool consistency, bleeding, and weight loss) after DSS treatment. Data are expressed as mean ± SEM (*n* = 5–7). *p<0.05 vs control of same dietary Pi group; ^#^*p*<0.05 vs Pi: 0.5% + DSS group; ^†^*p*<0.05 vs Pi: 1.0% + DSS group.

First, we observed the clinical symptoms including body weight loss and stool consistency and bleeding caused by colitis after DSS administration. All DSS-treated groups developed signs of colitis, such as weight loss and diarrhea during the study period. Body weight was significantly decreased in all DSS-treated groups and these changes were more severe in dose-dependent effect of Pi intake (Fig. 1A). Although there was no significant difference in food intake among the different Pi diets, it was significantly lower in the DSS-treated groups compared to the groups not receiving DSS (Fig. 1B). Furthermore, the high Pi diet (1.5%) significantly aggravated the DAI score in rats treated with DSS (Fig. 1C).

### Effects of dietary Pi on histopathology and mucin expression in DSS-induced colitis

**Fig. 2:**
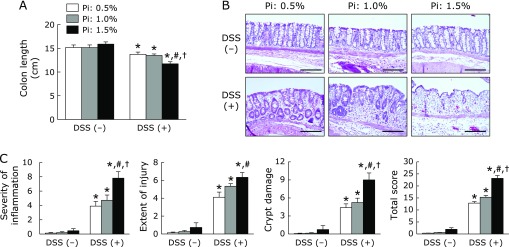
Increase in dietary Pi intake aggravated histopathology findings in DSS-induced colitis. (A) Changes in colon length. (B) Hematoxylin and eosin staining of colonic tissue. Magnification bar, 200 µm. (C) Histological scores (severity of inflammation, extent of injury, crypt damage, and total score) after DSS treatment. Data are expressed as mean ± SEM (*n* = 5–7). *p<0.05 vs control of same dietary Pi group; ^#^*p*<0.05 vs Pi: 0.5% + DSS group; ^†^*p*<0.05 vs Pi: 1.0% + DSS group.

**Fig. 3:**
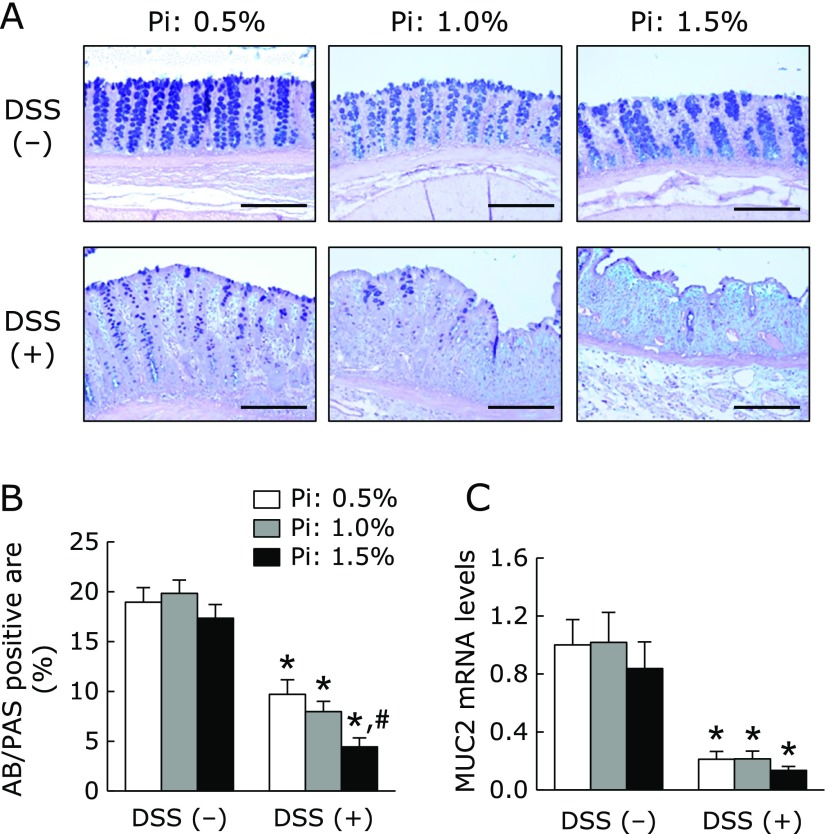
Effects of dietary Pi on colonic mucus secretion in DSS-induced colitis. (A) AB/PAS staining of colonic tissue in DSS-treated rat. Magnification bar, 200 µm. (B) Proportion of AB/PAS positive area in the colonic section of DSS-treated rat. Three sections randomly chosen from each rat were analyzed. (C) Mucin 2 (MUC2) mRNA levels of colonic tissue in DSS-induced rats were determined by quantitative real-time RT-PCR analysis. Data are expressed as mean ± SEM (*n* = 4–7). *p<0.05 vs control of same dietary Pi group; ^#^*p*<0.05 vs Pi: 0.5% + DSS group.

Next, we assessed the severity of DSS-induced colitis macroscopically. As shown in Fig. 2A, the colon length shortened by DSS treatment was more severe in the high Pi diet (1.5%) groups. The severity of colonic inflammation was further evaluated by histopathologic observations. As shown in Fig. 2B, DSS treatment induced destruction of epithelial architecture with a loss of crypts and epithelial integrity, submucosal edema, and infiltration of inflammatory cells. In particular, the high Pi diet (1.5%) group had more severe colonic inflammation and epithelial damage. These histologic observations were scored using criteria for histologic scoring of colitis as described above. Dietary Pi significantly exacerbated all histological scores in DSS-treated group in a dose-dependent manner (Fig. 2C). Furthermore, AB/PAS staining identified a significantly reduced number of mucous-secreting goblet cells in DSS-treated groups receiving a high Pi diet, especially 1.5% Pi group (Fig. 3A and B). Mucin 2 (MUC2) mRNA expression synthesized by the goblet cells in the colon was evaluated. Pi treatment did not affect colonic MUC2 mRNA expression in either the presence or absence of DSS (Fig. 3C).

### Effect of dietary Pi on colonic inflammation in DSS-induced colitis

**Fig. 4:**
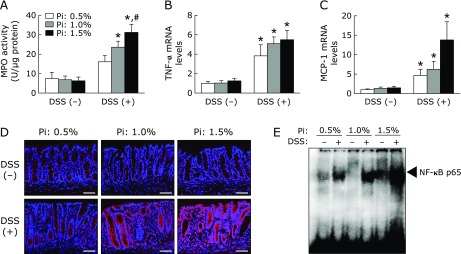
Increase in dietary Pi intake enhanced colonic inflammation through the activation of NF-κB in DSS-induced colitis. (A) MPO activity in the colonic tissue of DSS-induced rat. (B, C) Colonic inflammatory cytokine/chemokine mRNA levels in DSS-induced rats were determined by quantitative real-time RT-PCR analysis. (D) NF-κB p65 expression of colonic tissue was analyzed by immunofluorescent staining. Magnification bar, 300 µm. (E) NF-κB activity of colonic nuclear extract was evaluated by EMSA. Data are expressed as mean ± SEM (*n* = 4–7). *p<0.05 vs control of same dietary Pi group; ^#^*p*<0.05 vs Pi: 0.5% + DSS group; ^†^*p*<0.05 vs Pi: 1.0% + DSS group.

MPO activity in the colonic tissue was measured to evaluate inflammatory status. MPO activity was significantly higher in DSS treated groups; it was more severe in Pi intake group in a dose-dependent effect of Pi intake (Fig. 4A). We also measured colonic tumor necrosis factor-α (TNF-α) and monocyte chemottractant protein-1 (MCP-1) mRNA expression, which have been well characterized as a pivotal inflammatory cytokine/chemokine during colonic inflammation. As shown in Fig. 4B and C, TNF-α and MCP-1 mRNA levels were significantly increased in the DSS-treated group and they tended to be higher in the high Pi diet (1.5%) group in the presence of DSS.

To examine the ability of dietary Pi to stimulate inflammatory cytokine induction through an NF-κB-mediated pathway, we analyzed NF-κB expression and activation by immunofluorescence staining and EMSA, respectively. Immunofluorescence staining showed that NF-κB p65 was strongly expressed in intestinal epithelial cells and infiltrating inflammatory cells of inflamed colon in the DSS-treated group, and its expression was increased in high Pi diet (P: 1.5%) groups. Consistent with proinflammatory cytokine expression, EMSA analysis demonstrated NF-κB activity was dose-dependently increased in colonic nuclear extracts of DSS-induced colitis rats.

### Effect of Pi loading on inflammatory response in RAW264.7 cells

**Fig. 5:**
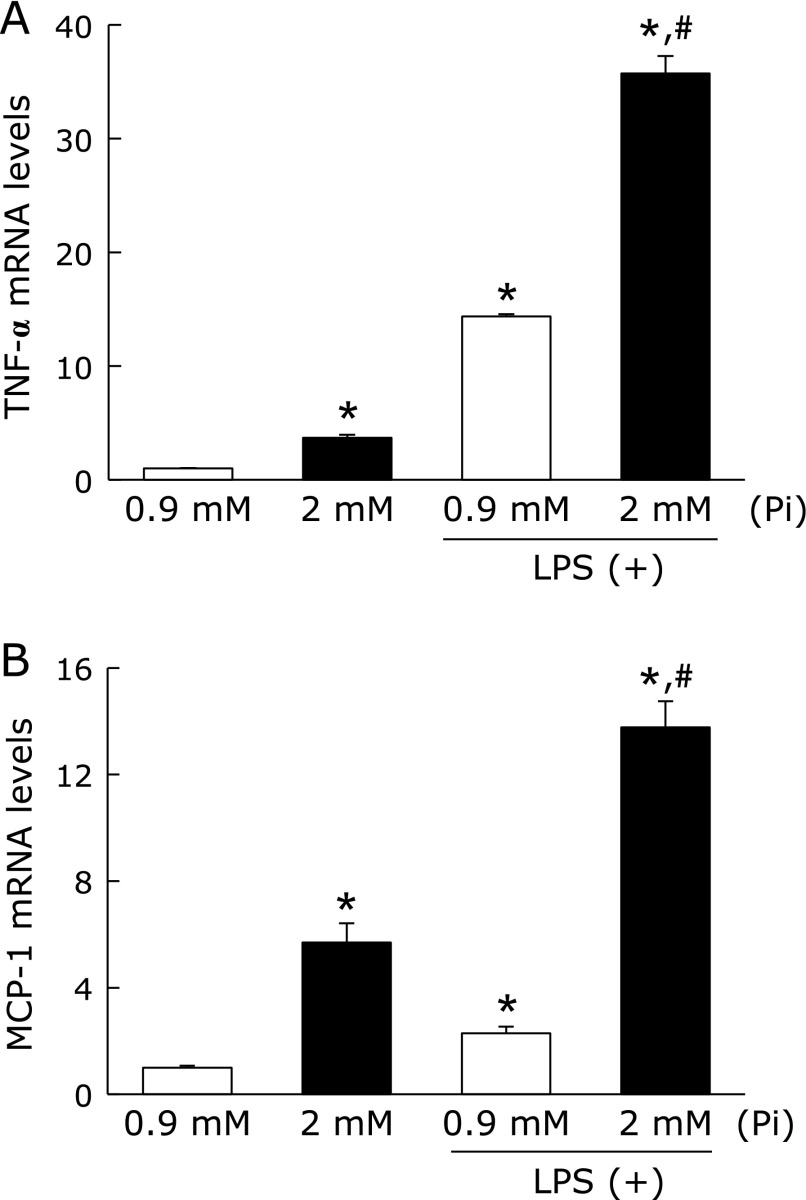
High Pi loading enhanced inflammatory response in LPS-stimulated RAW264.7 cells. RAW264.7 cells were preincubated with Pi (2 mM) for 12 h, followed by an incubation with LPS (1 µg/µl) for 1 h. Total RNA was extracted and relative TNF-α (A) and MCP-1 (B) mRNA levels was determined by quantitative real-time RT-PCR. Data are expressed as mean ± SEM (*n* = 3). *p<0.05 vs Pi: 0.9 mM; ^#^*p*<0.05 vs Pi: 0.9 mM + LPS.

**Fig. 6:**
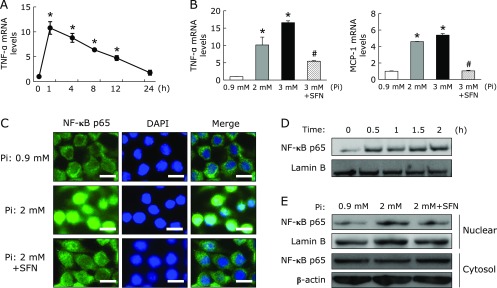
High Pi loading directly increased cytokine/chemokine mRNA expression through the activation of NF-κB in RAW264.7 cells. (A) RAW264.7 cells were treated with Pi (2 mM) for the indicated times (*n* = 3). (B) RAW264.7 cells were pretreated with SFN (10 µM) for 1 h, followed by treatment with different Pi concentrations for 1 h (*n* = 3). Relative TNF-α and MCP-1 mRNA levels were determined by quantitative real-time RT-PCR. (C) RAW264.7 cells were pretreated with SFN (10 µM) for 1 h and then exposed to Pi (2 mM) for 30 min. Cells were fixed, permeabilized and stained with NF-κB p65 and DAPI. Magnification bar, 10 µm. (D) RAW264.7 cells were treated with Pi (2 mM) for the indicated times and nuclear protein was extracted. Nuclear NF-κB p65 expression was determined by Western blot analysis. (E) RAW264.7 cells were pretreated with SFN (10 µM) for 1 h, and then treated with Pi (2 mM) for 1 h. Nuclear and cytosol protein was extracted and NF-κB p65 expression was detected by Western blot analysis. Data are expressed as mean ± SEM. *p<0.05 vs Pi: 0.9 mM; ^#^*p*<0.05 vs Pi: 3 mM.

To elucidate the impact of Pi on the inflammatory response of macrophages, the effects of Pi on cytokine/chemokine mRNA expression were evaluated by *in vitro* experiments using RAW264.7 macrophages. First, these cells were incubated with Pi for 12 h, and then exposed to LPS for 1 h. As a result, Pi loading synergistically increased LPS-stimulated TNF-α and MCP-1 mRNA expression in RAW264.7 cells (Fig. 5). Interestingly, in the absence of LPS, treatment of RAW264.7 cells with Pi significantly increased cytokine/chemokine mRNA expression. Thus, we also examined time- and dose-dependent effects of Pi on the expression of cytokine/chemokine to determine whether Pi directly causes an inflammatory response in the absence of LPS. RAW264.7 cells were treated with Pi in the absence of LPS for up to 24 h. TNF-α mRNA levels significantly increased within 1 h, and then declined to basal levels at 24 h. Furthermore, the effect of Pi on TNF-α and MCP-1 mRNA expression exhibited dose dependency (Fig. 6A and B), indicated that high Pi loading directly enhanced these cytokine/chemokine gene expressions. Next, we evaluated whether Pi loading directly stimulates proinflammatory cytokine induction through the NF-κB-mediated pathway. Since the nuclear translocation of the NF-κB p65 is a critical step for the activation of NF-κB signaling pathway, we investigated the impact of Pi on nuclear translocation of the NF-κB p65 by immunofluorescence staining and Western blot analysis. As expected, immunofluorescence staining showed that Pi-induced the transfer of NF-κB p65 into the nucleus, whereas untreated cells did not cause NF-κB p65 translocation (Fig. 6C). A similar result was acquired by Western blot analysis with nuclear extracts (Fig. 6D and E). To determine whether NF-κB activation was a prerequisite for proinflammatory cytokine induction, the effect of NF-κB inhibition on RAW264.7 cells was examined. Sulforaphane (SFN), which is an aliphatic isothiocyanate and known anti-inflammatory compound, inhibited LPS-induced NF-κB activation in RAW264.7 cells.^(28)^ SFN pretreatment inhibited Pi-induced TNF-α and MCP-1 mRNA expressions (Fig. 6B). Similarly, Pi-induced nuclear translocation of NF-κB p65 was also suppressed by SFN in RAW264.7 cells (Fig. 6C, D and E). Thus, NF-κB activation was required for the induction of inflammatory cytokine in Pi-stimulated RAW264.7 cells.

### Effect of Pi loading on ROS production in RAW264.7 cells

**Fig. 7:**
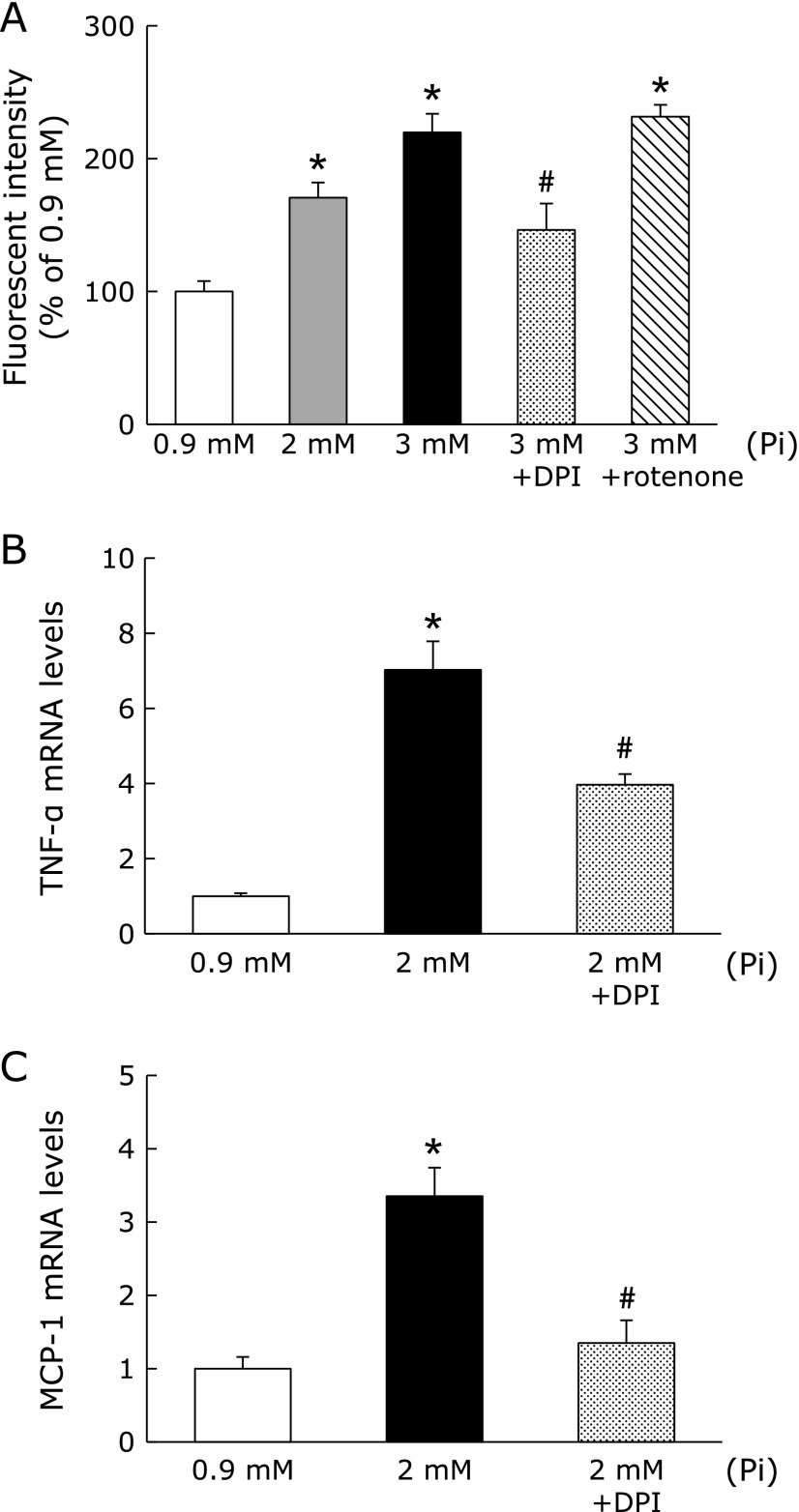
High Pi loading enhanced ROS production in RAW264.7 cells. (A) After incubation with APF (20 µM) for 30 min, cells were treated by different concentrations of Pi for 2 h with or without various oxidase inhibitors. (B, C) RAW264.7 cells were pretreated with DPI (10 µM) for 30 min, and then treated with Pi (2 mM) for 1 h. Data are expressed as mean ± SEM (*n* = 5). *p<0.05 vs Pi: 0.9 mM; ^#^*p*<0.05 vs Pi: 2 mM.

Since the activation of NF-κB was mediated by ROS,^(29)^ we evaluated whether high Pi loading could enhance ROS production. We determined ROS production using APF, which is a specific fluorescence probe for ROS. Pi loading significantly increased the ROS production in RAW264.7 cells in a dose-dependent manner (Fig. 7A). It is reported that Pi-induced ROS is produced mainly by the activation of NAD(P)H oxidase and mitochondrial respiratory chain. Thus, we next examined the effect of specific oxidase inhibitors on Pi-mediated ROS production. Fig. 7A showed that diphenyl iodonium (DPI), which is a specific inhibitor of NAD(P)H oxidase, suppressed Pi-induced ROS production. On the other hand, rotenone, an inhibitor of mitochondrial complex I could not inhibit ROS production. Furthermore, we examined whether inhibition of ROS production could suppress the inflammatory response in RAW264.7 cells. Pretreatment of DPI followed by Pi loading significantly inhibited Pi-induced TNF-α and MCP-1 mRNA expression (Fig. 7B and C). Therefore, Pi-mediated ROS generation could contribute to proinflammatory cytokine/chemokine induction in RAW264.7 cells.

## Discussion

To the best of our knowledge, the present study is the first to demonstrate that high Pi intake exacerbated intestinal inflammation in experimental colitis and high Pi loading increased proinflammatory cytokine expression through ROS production in RAW264.7 macrophages. Furthermore, previous epidemiologic, animal, and clinical studies showed that various nutrients could promote or prevent intestinal inflammation.^(2)^ Indeed, our findings suggest that excessive dietary Pi is one of the nutrients promoting intestinal inflammation.

Recent widespread consumption of Western diets and processing foods is associated with excessive intake of Pi.^(6–8)^ Moreover, previous studies reported that consumption of high Pi foods (e.g., such as fast foods and processed meat) increased the onset and relapse risk for IBD.^(3–5)^ However, it remains unclear whether dietary Pi intake is directly correlated to IBD, as the available databases used to estimate dietary Pi intake are inaccurate, and thus the Pi content of foods is significantly underestimated.^(8)^ Therefore, the present study was performed to examine the impact of dietary Pi on DSS-induced experimental colitis, which mimics ulcerative colitis-like disease. After DSS administration, intestinal inflammation with increased histological scores, NF-κB activation, and proinflammatory cytokine levels were observed and dietary Pi enhanced intestinal inflammation in a dose-dependent manner. Moreover, Pi acts as an important mediator that stimulates proinflammatory cytokine/chemokine in RAW264.7 macrophages, supporting the hypothesis that tissue resident and recruiting macrophages are the major cell types responsible for adverse effects of Pi on intestinal inflammation.

Several sodium-dependent Pi transporters and Pi uptake were observed in RAW264.7 cells,^(30,31)^ and it is presumed that Pi uptake into RAW264.7 cells results in the activation of macrophages. Oxidative stress has been linked to the pathogenesis of various diseases including IBD.^(32)^ Our study demonstrated that high Pi loading directly enhanced ROS production in RAW264.7 cells. In addition, inhibition of ROS generation using inhibitor of NAD(P)H oxidase partially suppressed Pi-induced inflammatory response. Therefore, Pi-induced ROS production is a possible reason for the activation of RAW264.7 macrophages. In contrast to NAD(P)H oxidase, mitochondrial ROS may only play a partial role in activation of RAW264.7 macrophages. Our results agreed with previous reports by ourselves and others showing that Pi loading increased ROS production, thus leading to calcification and endothelial dysfunction in a number of cell types.^(11–15)^

Pi homeostasis is tightly regulated by controlling intestinal and renal epithelial transport systems. Dietary Pi is absorbed in the proximal small intestine by subtype of type IIb sodium dependent cotransporters (NaPi-IIb) expressed on the apical membranes of enterocytes; an estimated 70% of all dietary Pi content is absorbed via the small intestine.^(33,34)^ This Pi transport was regulated by various factors, such as Pi intake,^(35)^ vitamin D_3_,^(36)^ and hormones.^(37,38)^. In addition to these factors, intestinal Pi transport is also affected by intestinal inflammation. Chen *et al.*^(39)^ reported that intestinal Pi absorption and NaPi-IIb expression were decreased in an experimental colitis model, and they were regulated by TNF-α. Therefore, high Pi intake and reduction of intestinal Pi absorption may increase luminal Pi concentrations in the colon. It is widely recognized that the small intestine is responsible for most Pi absorption, but Pi transport is also observed in the colon.^(34,40,41)^ Although the exact mechanism remains unclear, increased colonic Pi absorption may accelerate DSS-induced colonic inflammation.

In contrast, serum Pi levels were independently associated with inflammatory markers in CKD patients,^(19)^ and low-Pi diets could ameliorate systemic and renal inflammation in a CKD rat model.^(14,42)^ Moreover, recent studies demonstrated that elevated parathyroid hormone and fibroblast growth factor 23, known as phosphaturic hormones,^(6)^ can directly stimulate hepatic production of inflammatory cytokines,^(43,44)^ and these were positively correlated to inflammatory markers in CKD patients.^(45,46)^ Therefore, we speculate that a high luminal Pi concentration in the colon may enhance colonic inflammation, whereas high Pi intake-induced systemic alteration in phosphate-regulating hormones can also affect sensitivity to DSS-induced colitis. Since we could not determine which factors are important for intestinal inflammation, further investigation is required.

In clinical practice, the harmful effects of Pi should be taken into account. Colonoscopy establishes the diagnosis and subsequent follow-up plan in IBD patients, and oral sodium Pi is widely used as a colonic cleansing agent for colonoscopy. However, oral sodium Pi use may be associated with development of colonic mucosal abnormalities due to acute Pi overloading.^(47,48)^ Therefore, sodium Pi solutions should probably not be used in IBD patients due to the high risk of toxicity on the intestine. Importantly, these agents contain large amounts of Pi (more than 10 times amount the usual daily Pi dietary intake). On the other hand, the 0.5% Pi diet was designated as the control in this study and high Pi diets contain more than two or three times that amount. Therefore, a high Pi diet can enhance intestinal inflammation within the range that can be routinely ingested. Furthermore, in terms of therapeutic strategies, excessive Pi intake should be avoided and a low Pi diet may be beneficial to suppress intestinal inflammation. Since disease activity is correlated with nutritional status,^(49)^ dietary management including phosphate intake is also necessary for improvement of inflammatory status in IBD patients.

In conclusion, our results provide new insights into a dietary therapeutic strategy for IBD based on observations that excessive dietary Pi exacerbated intestinal inflammation through the production of proinflammatory cytokines/chemokines from activated macrophages. Further epidemiological and clinical studies are required to clarify how dietary Pi is directly associated with pathogenesis and the clinical condition of IBD patients.

## Figures and Tables

**Table 1:** Composition of the experimental diets

	Pi: 0.5% (g)	Pi: 1.0% (g)	Pi: 1.5% (g)
AIN-93G	59.5	59.5	59.5
Milk casein	20	20	20
Sugar	4.77	4.77	4.77
Mineral Mix	1.56	1.56	1.56
CaCO_3_	1.26	1.26	1.26
Soy bean oil	7	7	7
KH_2_PO_4_	1.52	3.71	5.91
KCl	2.41	1.2	0
Dextrin	1.99	0.99	0
Total	100	100	100

We used a mineral mix and an altered AIN-93G diet without casein, CaCO_3_, and KH_2_PO_4_.

**Table 2:** Sequence of oligonucleotide primers in the quantitative RT-PCR analysis

Gene name	Forward	Reverse
rat TNF-α	5'-GTCGTAGCAAACCACCAAGC-3'	5'-TGTGGGTGAGGAGCACGTAG-3'
rat MCP-1	5'-CCCTAAGGACTTCAGCACCTTTG-3'	5'-AAGTGCTTGAGGTGGTTGTGG-3'
rat MUC2	5'-CATCAAAGGTGGTGATGTGG-3'	5'-AGCTGCACGGACACCTCTAT-3'
rat GAPDH	5'-AGTTCAACGGCACAGTCAAG-3'	5'-GTGGTGAAGACGCCAGTAGA-3'
mouse TNF-α	5'-AGCCTGTAGCCCACGTCGTA-3'	5'-TCTTTGAGATCCATGCCGTTG-3'
mouse IL-1β	5'-GTGGACCTTCCAGGATGAGG-3'	5'-CGGAGCCTGTAGTGCAGTTG-3'
mouse MCP-1	5'-CCCAATGAGTAGGCTGGAGA-3'	5'-TCTGGACCCATTCCTTCTTG-3'
mouse β-actin	5'-CTGGGGTGTTGAAGGTCTCAAACATG-3'	5'-CTGACCCTGAAGTACCCCATTGAACA-3'
